# Sociodemographic Disparities in Atopic Dermatitis Prevalence in Spain

**DOI:** 10.3390/jcm15114289

**Published:** 2026-06-01

**Authors:** Lucía Cayuela, Rocío C. Bueno-Molina, Juan-Carlos Hernández-Rodríguez, Mercedes Sendín-Martín, José-Juan Pereyra-Rodríguez, Aurelio Cayuela

**Affiliations:** 1 Department of Internal Medicine, Hospital Severo Ochoa, 28911 Leganés, Spain; luccayrod@gmail.com; 2 Department of Dermatology, Virgen del Rocío University Hospital, 41013 Seville, Spain; j.carlos.her.rod@gmail.com (J.-C.H.-R.); mercedessendin@gmail.com (M.S.-M.); jpereyra@us.es (J.-J.P.-R.); 3 Department of Medicine, University of Seville, 41004 Seville, Spain; 4 Independent Researcher, 41013 Seville, Spain; aurelio.cayuela@gmail.com

**Keywords:** dermatitis, atopic, prevalence, socioeconomic factors, urban population, sex factors, Spain

## Abstract

**Background**: Atopic dermatitis (AD) is a widespread, chronic inflammatory skin condition with substantial global impact. While regional studies have explored its prevalence in Spain, comprehensive national data detailing AD prevalence across various sociodemographic groups remain limited. Objective: This study aimed to estimate the 2023 prevalence of AD in Spain and explore disparities across key sociodemographic groups. **Methods**: We conducted a retrospective cross-sectional study using Spain’s National Health System Primary Care Clinical Database (BDCAP). We identified AD cases via ICPC code S87. We calculated crude and age-standardized prevalence rates (ASPRs) and rate ratios, stratified by sex, age, country of origin, municipality size, income, and employment status. The 2013 European Standard Population was used for age standardization. **Results**: In 2023, an estimated 2,706,675 individuals in Spain were living with AD, with women (1,451,216 cases) experiencing a higher burden than men (1,255,459 cases), and also presenting with a higher mean age (27.23 years vs. 22.96 years for men). Urban living showed a clear dose–response relationship with AD prevalence. In cities with over 500,000 inhabitants, ASPRs reached 7.74% in men and 8.52% in women; significantly higher than in rural areas, where rates were 5.66% and 6.57%, respectively. Individuals in the middle-income bracket (€18,000–€99,999) consistently exhibited the highest prevalence, and the non-active employment group also demonstrated an elevated risk (ASPRs: 6.51% for men, 7.28% for women). Finally, Spanish-born individuals generally had higher AD prevalence compared to most foreign-born populations. **Conclusions**: Atopic dermatitis in Spain displays significant sociodemographic disparities, with urban environments, female sex, and non-active employment status emerging as key risk factors. These findings underscore the importance of targeted public health interventions and equity-focused dermatological planning.

## 1. Introduction

Atopic dermatitis (AD) is a chronic inflammatory skin disease characterised by itching, dry skin, and recurrent eczematous lesions. It results from interactions between genetic susceptibility, immune dysregulation, environmental triggers, and skin barrier dysfunction. Increasing evidence also highlights the role of skin microbial dysbiosis, particularly reduced microbial diversity and Staphylococcus aureus colonisation, in driving disease severity and flare-ups [1,2,3].

Globally, AD is one of the most common non-fatal diseases, placing a notable burden on healthcare systems, particularly in high-income countries and among children [4,5]. Prevalence estimates vary by region and methodological approach, typically ranging from 15% to 30% in children and 2% to 10% in adults [6,7,8,9,10,11]. Higher rates are often observed in females, young children, and urbanised areas [5].

In Spain, regional studies confirm a substantial prevalence of AD. For example, a population-based study in Aragón [12] found a 15.5% prevalence among children, particularly girls aged 3 to 9 years. In adults, a study from Catalonia [13] reported a prevalence of 8.7%, while research in Valencia showed a 10.6% prevalence among infants in their first year of life [14]. These findings align with broader European data [6,11,15], where childhood prevalence ranges from 0.96% to 22.6%, and adult prevalence ranges from 1.2% to 17.1%.

Despite these findings, national-level data stratified by sociodemographic characteristics remain limited. Most available studies are regional and provide fragmented insights. While some evidence links AD with urban living and humid climates, associations with socioeconomic status and rural versus urban residence remain inconsistent, especially in children [12,16].

The National Health System Primary Care Clinical Database (BDCAP) offers a robust source of standardised electronic health record data from primary care centres across Spain’s Autonomous Communities. It includes detailed information on diagnoses, prescriptions, referrals, and comorbidities, and has been widely used in health research, including studies in dermatology [17].

This study estimates the 2023 prevalence of AD in Spain using BDCAP data, stratified by key sociodemographic factors, to inform national public health strategies and guide clinical planning.

## 2. Materials and Methods

### 2.1. Study Design and Data Sources

We conducted a retrospective cross-sectional study to estimate the prevalence of atopic dermatitis (AD) in Spain in 2023. Data were drawn from the National Health System Primary Care Clinical Database (BDCAP), an anonymised national database that compiles standardised clinical information annually from electronic health records across Spanish primary care [1]. It includes all health problems marked as “active” during the year, covering both new diagnoses and ongoing chronic conditions. Diagnoses are coded using the International Classification of Primary Care (ICPC), allowing consistent classification of conditions across regions [2].

### 2.2. Study Population and Case Definition

The study population included all individuals with at least one active or open health problem recorded in BDCAP in 2023. This “treated population” was selected as the denominator for prevalence estimation because the BDCAP database captures information exclusively for individuals who actively utilise primary care services within a given year. Using the total census population as the denominator would introduce substantial bias by artificially reducing prevalence estimates, as it would include individuals who have relocated, exclusively use private healthcare services, or did not access healthcare during 2023 [3]. Accordingly, the resulting estimates should be interpreted as period prevalence within a healthcare-attending population rather than prevalence in the general population [2].

Prevalent cases of AD were identified using ICPC code S87 (Atopic Eczema/Dermatitis), recorded as active or open in 2023. While S87 constitutes the standardised diagnostic code across primary care centres in Spain, it encompasses a broad clinical spectrum. Consequently, some degree of heterogeneity in diagnostic classification and inter-practitioner variability is inevitable, including potential overlap between classic atopic dermatitis and other eczematous conditions.

### 2.3. Data Collection and Variables

Data on AD cases were stratified by sociodemographic and clinical characteristics. Sex was recorded as male or female. Age was grouped into 5-year intervals, from 0–4 years up to 95 years and older. Country of origin was classified using a modified version of World Health Organization regional groupings tailored to the Spanish context [4], including Spain, the European Union (excluding Spain), the Eastern Mediterranean region, Latin America, and Other or Unknown, which included individuals from Asia, the Pacific, North America, other European countries, and African countries outside the Eastern Mediterranean.

Municipality size was based on the population of the area served by the patient’s health centre and grouped into five categories: up to 10,000; 10,001 to 50,000; 50,001 to 100,000; 100,001 to 500,000; and over 500,000 inhabitants. Income level was categorised as very low (no employment income), less than €18,000, between €18,000 and €99,999, greater than €100,000, or unclassified in cases of missing data. Employment status was recorded as active, unemployed, pensioner, non-active (including children and dependents), or other, which included individuals covered by mutual insurance schemes. The “non-active” group represents a highly heterogeneous and strongly age-dependent category that contains a high concentration of paediatric individuals.

### 2.4. Statistical Analysis

Descriptive statistics were used to summarise AD cases by sociodemographic group, reporting absolute denominator population sizes alongside crude counts and percentages to enhance transparency regarding sample distributions. Age-standardised prevalence rates (ASPRs) per 100 individuals were calculated using direct standardisation, with the 2013 European Standard Population as the reference [18]. Age-specific rates were weighted and summed to produce ASPRs [5]. Ninety-five per cent (95% CIs) for the ASPRs were calculated using the standard analytical formula for the variance of a directly standardised rate, assuming a normal distribution, to assess the precision of the estimate. Rate ratios (RRs) and their corresponding 95% CIs were calculated to compare AD prevalence across sociodemographic categories, using a reference group [6]. All analyses were stratified by sex.

Given the cross-sectional nature of the study design, all investigated sociodemographic indicators, such as municipality size, income tier, and employment designation, are interpreted strictly as associated clinical characteristics rather than causal risk factors or predictive determinants.

### 2.5. Ethical Considerations

This study adhered to the Declaration of Helsinki and STROBE guidelines (Table A1). Data were fully anonymised and obtained from the Spanish National Health System’s Statistical Portal (BDCAP). According to Spanish law (Law 14/2007), the use of anonymised secondary data does not require patient consent or ethics committee approval.

## 3. Results

In 2023, a total of 1,255,459 men and 1,451,216 women in Spain were identified as having prevalent AD, confirming a higher overall prevalence among women.

**Figure 1:**
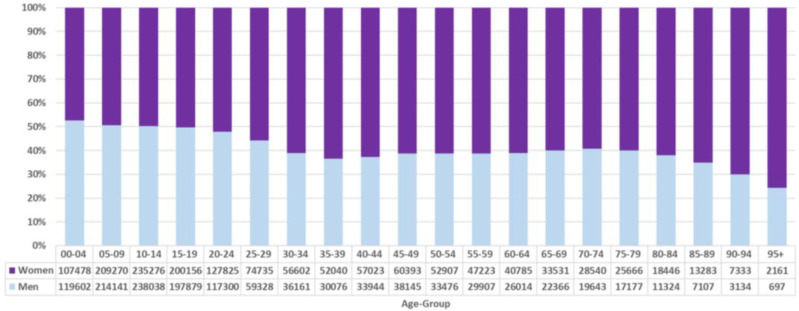
Age and sex distribution of atopic dermatitis prevalent cases in Spain in 2023.

**Figure 2:**
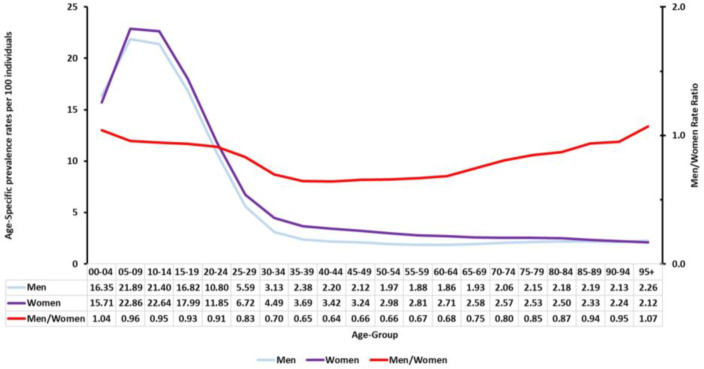
Age-specific prevalence rates of atopic dermatitis per 100 individuals, stratified by sex, and male-to-female rate ratio across age groups in Spain (2023).

Clear sex-based differences in age distribution were observed (Table 1). The mean age among women with AD was 27.23 years (95% CI: 27.20–27.27), notably higher than that of men, whose mean age was 22.96 years (95% CI: 22.92–23.00). Stratified age group analysis (Figure 1) showed that male cases predominated during childhood and adolescence, whereas female cases were more prevalent from the age of 25 onwards. These patterns are further illustrated in Figure 2, which displays age-specific prevalence rates and male-to-female ratios across the lifespan.

A positive association between municipality size and AD prevalence was evident in both sexes (Table 2 and Table 3). Compared to individuals living in municipalities with fewer than 10,000 inhabitants (reference group), those residing in increasingly larger urban areas had higher crude and age-standardised rate ratios (RRs) for AD. While age adjustment slightly attenuated these associations, the trend remained robust, indicating that urbanicity exhibits a strong and independent association with AD prevalence, regardless of sex.

Income showed a nonlinear relationship with AD prevalence (Table 2 and Table 3). Individuals in middle-income groups (<€18,000/year and €18,000–99,999/year) had modestly elevated RRs compared to the very low-income reference group, in both men and women. Age standardisation amplified these associations, suggesting an independent link between intermediate income brackets and recorded disease prevalence. Conversely, the unclassified income group consistently showed significantly lower RRs, even after adjustment, across both sexes.

The non-active population exhibited the highest crude prevalence and RRs compared to active individuals (reference group), in both men and women (Table 2 and Table 3). Although age adjustment substantially reduced the RRs for this group, they remained the category with the highest recorded prevalence. However, because this administrative category holds a naturally high concentration of paediatric individuals, this finding heavily reflects underlying age structures rather than an independent socio-occupational liability. Other employment categories, such as pensioners, the unemployed, and other situations, generally showed lower or near-parity RRs post-adjustment, with pensioners experiencing a slight increase in adjusted RRs.

Compared to individuals born in Spain, foreign-born individuals consistently exhibited lower crude RRs for AD (Table 2 and Table 3). However, after age standardisation, RRs for all non-Spanish groups increased, narrowing the disparity. In both sexes, the “Other or Unknown” group was the only one to exceed an RR of 1 post-adjustment, indicating comparable or higher prevalence rates than native-born individuals. These findings suggest that differences in population age structures between native and migrant groups largely account for the crude disparities.

## 4. Discussion

This nationally representative study provides age-standardised estimates of AD prevalence in Spain for 2023, stratified by key sociodemographic factors. Our findings reveal significant disparities associated with urban living, income level, employment status, sex, and country of birth, underscoring how structural and environmental conditions are linked to the recorded AD burden. These insights are critical for informing targeted public health responses and equitable dermatological care.

A key finding of this study is the significantly higher prevalence of AD in larger municipalities. Individuals residing in cities with more than 500,000 inhabitants exhibited the highest age-standardised prevalence rates (ASPRs), reaching 7.74% in men and 8.52% in women, compared with 5.66% and 6.57%, respectively, in rural areas. This pattern is consistent with the well-described “urbanisation effect”, which has been widely hypothesised to stem from environmental and lifestyle exposures characteristic of urban settings, including increased exposure to air pollutants (e.g., NO_2_, PM_2.5_), reduced microbial diversity, and factors associated with modern living that may contribute to skin barrier dysfunction and immune dysregulation [4,19].

Supporting this interpretation, large-scale epidemiological studies and meta-analyses have reported an increased risk of AD among individuals living in urban environments, with relative risks ranging from 1.35 to 1.95 [19,20,21]. Additional exposures such as psychosocial stress, indoor allergens, and dietary patterns may also contribute to disease development and exacerbation, while neighbourhood-level socioeconomic conditions can further influence risk and severity [4,22].

Importantly, the observed association persisted after age standardisation, suggesting a potential independent relationship with urban residence. However, in the absence of direct measures of environmental exposures (e.g., air quality, microbial diversity, or lifestyle factors), these mechanisms should be presented as plausible explanatory hypotheses rather than causal conclusions.

Consistent with global epidemiological patterns, women in this study exhibited higher AD prevalence than men across nearly all age and sociodemographic strata, with the disparity most pronounced in adulthood. This finding reflects the well-described sex reversal in AD epidemiology, whereby males predominate in childhood, but females show higher prevalence in later life [23,24,25]. These differences are likely multifactorial, involving hormonal, immunological, behavioural, and sociocultural determinants [26]. Moreover, adult women with AD have been reported to experience a greater quality-of-life burden and may be relatively undertreated compared with men [27].

The association between income and AD was complex and nonlinear. While a higher proportion of AD cases were found in women with lower incomes, the highest ASPRs occurred in the middle-income group (€18,000–99,999) for both sexes. This complexity reflects the mixed evidence on the relationship between socioeconomic position and atopic dermatitis found globally [28]. In high-income and industrialised countries, the prevalence of AD is generally higher, with individuals of a higher socioeconomic status often reporting higher prevalence due to increased awareness, healthcare access, and reporting [4,5]. However, some studies have shown that low household income is associated with a higher risk of doctor-diagnosed atopic dermatitis in children, potentially due to factors such as limited access to healthcare, increased psychosocial stress, or environmental exposures [29]. This may reflect a combination of environmental and behavioural exposures: higher-income individuals may experience increased contact with indoor allergens (consistent with the hygiene hypothesis), whereas lower-income groups may be more affected by housing quality, chronic stress, or barriers to healthcare access [28,30].

Spain’s universal healthcare system may buffer some of these disparities, ensuring baseline access to care and treatment [31]. Nonetheless, structural challenges remain, such as limited availability of dermatological products or treatment options in pharmacies located in low-income areas, which can perpetuate inequalities in disease burden [32].

The notably low prevalence among individuals with unclassified income is striking and may reflect data quality issues, non-disclosure, or unique patterns in healthcare utilisation, warranting further investigation.

Employment status was a strongly associated characteristic of AD prevalence. The “Non-active” group, including students, homemakers, and those unable to work, had the highest ASPRs (men: 6.51%; women: 7.28%). Even after age adjustment, this group remained at elevated risk, suggesting a potential dual burden of chronic illness and socioeconomic vulnerability. Employment status affects AD prevalence primarily through its association with socioeconomic status, occupational exposures, and work-related stress [30,33]. Studies have shown that individuals with higher socioeconomic status, often correlating with employment in professional or technical occupations, report higher AD prevalence, while occupational exposures in high-risk jobs are associated with increased incidence and persistence of AD [34,35].

Pensioners also exhibited higher standardised rates compared to the active population (men: 3.42%; women: 4.37%), possibly reflecting the cumulative burden of chronic disease and immunosenescence. Interestingly, individuals in “Other situations” had the lowest ASPRs, and unemployed individuals showed similar or slightly lower rates than the active workforce. These patterns may reflect differing occupational exposures, stress levels, or environmental factors associated with workplace environments, consistent with existing research on occupational risks in AD [36,37].

Notable differences in AD prevalence were observed by country of birth. Spanish-born individuals and those in the “Other or Unknown” group consistently showed the highest ASPRs (e.g., women: 7.77% and 8.05%, respectively), while those born in Africa, Latin America, the EU (excluding Spain), and the Eastern Mediterranean exhibited markedly lower prevalence. For instance, men born in Africa had an ASPR of just 3.76%, roughly half the rate of Spanish-born men.

These disparities are consistent with global patterns showing that children born in low-prevalence countries who migrate to high-prevalence countries initially have lower rates of atopic dermatitis compared to native-born children, but their risk increases with longer residence [38,39]. There is wide variation in atopic dermatitis prevalence by country, with higher rates observed in high-income, industrialised, and urbanised countries, and lower rates in many low- and middle-income countries [7,14,40]. These disparities likely reflect a complex mix of genetic predisposition, environmental transitions, acculturation, and healthcare access. Migration can alter allergic disease risk depending on timing of migration, urbanisation, and exposure to allergens or irritants [41]. Lower recorded prevalence among foreign-born populations may reflect underdiagnosis or undercoding due to reduced healthcare utilisation and barriers to care, rather than true biological protection. Individuals with skin of colour may also face greater disease burden and diagnostic barriers, despite being underrepresented in dermatology research [42].

The elevated rates in the “Other or Unknown” category may result from data misclassification, but could also reflect specific high-risk migrant groups or individuals facing systemic challenges, such as limited healthcare access or heightened exposure to environmental stressors. These findings underscore the need for disaggregated, culturally-sensitive research and policies that account for the intersection of race, migration status, and socioeconomic position [43].

### Limitations

Several limitations should be acknowledged. The retrospective cross-sectional design precludes causal inference and limits the ability to attribute independent predictive effects to the examined sociodemographic factors. The use of primary care data (BDCAP) may introduce selection bias, as individuals with low healthcare utilisation or those managed exclusively in specialised settings are likely underrepresented.

Defining the denominator as the “treated population” captures period prevalence among healthcare-seeking individuals rather than a true census population, thereby limiting comparability with general population or survey-based estimates. Although ICPC coding is standardised, it may not fully reflect the clinical spectrum or severity of AD; notably, code S87 represents a broad diagnostic category (“Atopic Eczema/Dermatitis”), introducing potential diagnostic variability, classification heterogeneity, and misclassification with other eczematous conditions.

Additionally, broad sociodemographic categories, such as the heterogeneous and age-dependent “non-active” employment group, may obscure within-group differences. Finally, residual confounding is likely, as key biological and environmental factors (e.g., lifestyle, environmental exposures, microbiome, and genetics) were not available, and temporal trends could not be assessed.

Despite these limitations, the study identifies critical sociodemographic associations with AD in Spain and highlights persistent inequities by sex, income, employment, and migration status. Future research should adopt longitudinal designs to explore causality, disease trajectories, and the impacts of interventions.

Efforts should focus on disaggregating migrant populations, refining socioeconomic categorisations, and incorporating objective environmental measures (e.g., air quality, housing conditions, occupational exposures). Moreover, future studies must explore how gender, migration, and socioeconomic status intersect to shape AD outcomes.

Finally, research on the health effects of climate change, as well as intervention trials targeting urban environments, healthcare access, and social support, could inform effective public health responses to reduce the growing burden of AD.

## 5. Conclusions

This study reveals that atopic dermatitis in Spain remains strongly patterned by sociodemographic factors, including municipality size, income, employment status, and country of birth. Women, individuals living in large urban areas, and those in non-active employment categories bear the greatest burden of disease. Importantly, age standardisation revealed that many observed disparities are not simply due to age structure, but rather reflect underlying systemic or environmental differences.

Public health responses should focus on addressing urban exposures, occupational risk factors, and access to care, particularly in underserved populations. Future research should prioritise intersectional approaches—accounting for gender, migration, socioeconomic status, and race—to better understand and mitigate the unequal burden of AD in Spain.

## Figures and Tables

**Table 1:** Number and prevalence (per 100 individuals) of atopic dermatitis cases by sociodemographic and socioeconomic characteristics and sex, Spain, 2023.

	Men		Women	
	n	Prevalence (%)	n	Prevalence (%)
**Municipality Size**				
<10,000 residents	149,672	4.88	172,436	5.49
10,001–50,000 residents	389,885	5.98	445,479	6.47
50,001–100,000 residents	184,670	6.06	216,647	6.48
100,001–500,000 residents	314,786	6.55	366,481	6.88
>500,000 residents	216,444	7.00	250,173	7.02
**Income Level**				
Very low	183,139	5.93	237,766	5.08
<18,000 €/year	558,983	6.02	683,310	6.63
18,000–99,999 €/year	494,147	6.36	511,576	7.37
>100,000 €/year	15,898	6.04	15,014	7.92
Unclassified	3292	2.49	3007	2.53
**Employment Status**				
Active	274,788	3.19	362,561	4.52
Pensioner	100,920	2.26	125,539	2.78
Non-active	816,145	16.25	849,949	13.32
Unemployed	30,031	2.72	51,356	3.57
Other situations	33,494	2.49	61,761	3.24
**Country of Birth**				
Spain	1,063,214	6.76	1,203,197	7.12
Africa	5998	2.66	4700	4.44
Eastern Mediterranean	17,889	3.28	18,085	4.27
Latin America	40,278	3.49	66,598	4.00
European Union (EU)	20,033	2.85	27,926	3.51
Other or Unknown	108,047	4.97	130,709	5.49

**Table 2:** Age-standardised prevalence and rate ratios of atopic dermatitis by sociodemographic characteristics in Spanish men in 2023.

	ASPR	Confidence Interval	Rate Ratio	Confidence Interval
**Municipality Size**				
<10,000 residents	5.66	[5.63; 5.69]	1	
10,001–50,000 residents	6.22	[6.2; 6.24]	1.1	[1.09; 1.11]
50,001–100,000 residents	6.27	[6.24; 6.3]	1.11	[1.10; 1.12]
100,001–500,000 residents	7.03	[7.01; 7.06]	1.24	[1.24; 1.25]
>500,000 residents	7.74	[7.71; 7.77]	1.37	[1.36; 1.38]
**Income Level**				
Very low	5.19	[5.16; 5.21]	1	
<18,000 €/year	6.57	[6.55; 6.59]	1.27	[1.26; 1.27]
18,000–99,999 €/year	7.4	[7.38; 7.43]	1.43	[1.42; 1.43]
>100,000 €/year	6.26	[6.14; 6.39]	1.21	[1.19; 1.23]
Unclassified	1.89	[1.82; 1.96]	0.36	[0.35; 0.38]
**Employment Status**				
Active	3.24	[3.2; 3.28]	1	
Pensioner	3.42	[3.36; 3.48]	1.06	[1.05; 1.06]
Non-active	6.51	[6.48; 6.54]	2.01	[2.00; 2.02]
Unemployed	2.48	[2.42; 2.55]	0.77	[0.76; 0.78]
Other situations	2.97	[2.93; 3.02]	0.92	[0.91; 0.93]
**Country of Birth**				
Spain	6.83	[6.81; 6.84]	1	
Africa	3.76	[3.63; 3.89]	0.55	[0.54; 0.56]
Eastern Mediterranean	3.95	[3.88; 4.02]	0.58	[0.57; 0.59]
Latin America	4.15	[4.1; 4.2]	0.61	[0.6; 0.61]
European Union (EU)	4.42	[4.34; 4.49]	0.65	[0.64; 0.66]
Other or Unknown	7.2	[7.15; 7.24]	1.05	[1.05; 1.06]

ASPR: Age-Standardised Prevalence Rate.

**Table 3:** Age-standardised prevalence and rate ratios of atopic dermatitis by sociodemographic characteristics in Spanish women in 2023.

	ASPR	Confidence Interval	Rate Ratio	Confidence Interval
**Municipality Size**				
<10,000 residents	6.57	[6.54; 6.61]	1	
10,001–50,000 residents	7.12	[7.1; 7.14]	1.08	[1.08; 1.09]
50,001–100,000 residents	7.18	[7.15; 7.21]	1.09	[1.09; 1.1]
100,001–500,000 residents	7.98	[7.96; 8.01]	1.21	[1.21; 1.22]
>500,000 residents	8.52	[8.48; 8.55]	1.3	[1.29; 1.3]
**Income Level**				
Very low	6.00	[5.98; 6.03]	1	
<18,000 €/year	7.66	[7.64; 7.68]	1.28	[1.27; 1.28]
18,000–99,999 €/year	8.13	[8.1; 8.15]	1.35	[1.35; 1.36]
>100,000 €/year	6.68	[6.54; 6.82]	1.11	[1.09; 1.13]
Unclassified	1.63	[1.57; 1.69]	0.27	[0.26; 0.28]
**Employment Status**				
Active	4.25	[4.21; 4.28]	1	
Pensioner	4.37	[4.3; 4.45]	1.03	[1.02; 1.04]
Non-active	7.28	[7.26; 7.3]	1.72	[1.71; 1.72]
Unemployed	3.28	[3.2; 3.35]	0.77	[0.76; 0.78]
Other situations	3.8	[3.75; 3.85]	0.9	[0.89; 0.9]
**Country of Birth**				
Spain	7.77	[7.76; 7.78]	1	
Africa	5.02	[4.85; 5.18]	0.65	[0.63; 0.66]
Eastern Mediterranean	4.69	[4.61; 4.77]	0.60	[0.6; 0.61]
Latin America	4.96	[4.91; 5.01]	0.64	[0.63; 0.64]
European Union (EU)	5.02	[4.95; 5.1]	0.65	[0.64; 0.65]
Other or Unknown	8.05	[8; 8.1]	1.04	[1.03; 1.04]

ASPR: Age-Standardised Prevalence Rate.

## Data Availability

The data supporting the conclusions of this study are publicly available at https://pestadistico.inteligenciadegestion.sanidad.gob.es/publicoSNS/comun/DefaultPublico (accessed on 15 March 2026).

## Abbreviations

The following abbreviations are used in this manuscript:

AD	Atopic Dermatitis
BDCAP	Base de Datos Clínicos de Atención Primaria (National Health System Primary Care Clinical Database)
ICPC	International Classification of Primary Care
ASPR(s)	Age-Standardised Prevalence Rate(s)
RR(s)	Rate Ratio(s)
CI	Confidence Interval

## Appendix A

**Table A1:** STROBE Statement—Checklist of items that should be included in reports of observational studies (Adapted for Cross-Sectional Studies).

Item No.	Recommendation	Page/Paragraph No.
**Title and abstract**		
1 (a)	Indicate the study’s design with a commonly used term in the title or the abstract.	Abstract: Objective/Methods
1 (b)	Provide in the abstract an informative and balanced summary of what was done and what was found.	Abstract: All sections
**Introduction**		
2	Explain the scientific background and rationale for the investigation being reported.	Abstract: Background; Introduction (Section 1)
3	State specific objectives, including any prespecified hypotheses.	Abstract: Objective; Introduction (Section 1)
**Methods**		
4	Present key elements of study design early in the paper.	Methods (Section 2, para 1)
5	Describe the setting, locations, and relevant dates, including periods of recruitment, exposure, follow-up, and data collection.	Methods (Section 2, para 1)
6	Give the eligibility criteria, and the sources and methods of selection of participants.	Methods (Section 2, para 2)
7	For each variable of interest, give the sources of data and details of methods of assessment (measurement). Describe comparability of assessment methods if there is more than one group.	Methods (Section 2, para 3)
8	Describe any efforts to address potential sources of bias.	Discussion: Limitations (Section 4, last para)
9	Explain how the study size was arrived at.	Methods (Section 2, para 2—implicitly, as all eligible cases in database were included)
10	Describe all statistical methods, including those used to control for confounding.	Methods (Section 2, para 3)
11	Describe any sensitivity analyses.	N/A
**Results**		
12 (a)	Report the numbers of individuals at each stage of the study—e.g., numbers potentially eligible, examined for eligibility, confirmed eligible, included in the study, completing follow-up, and analysed.	Results (Section 3, para 1)
12 (b)	Give reasons for non-participation at each stage.	N/A (Database study, no participation stages)
12 (c)	Consider use of a flow diagram.	N/A
13 (a)	Give characteristics of study participants (e.g., demographic, clinical, social) and information on exposures and potential confounders.	Results (Section 3, para 1); Table 1
13 (b)	Indicate number of participants with missing data for each variable of interest.	Results (Table 1—implicit in ‘Unclassified’ and ‘Other/Unknown’ categories)
14	Report numbers of outcome events or summary measures of outcomes.	Results (Section 3, all paragraphs); Table 1
15 (a)	Give unadjusted estimates and, if applicable, confounder-adjusted estimates and their precision (e.g., 95% confidence interval). Make clear which confounders were adjusted for and why they were chosen.	Results (Section 3, all paragraphs, specifically ASPRs and RRs); Methods (Section 2, para 3—for age standardization)
15 (b)	Report category boundaries when continuous variables were categorized.	Results (Section 3—e.g., income categories); Table 1
15 (c)	If relevant, consider translating estimates of relative risk into absolute risk for a meaningful time period.	N/A (Cross-sectional prevalence study)
16	Report other analyses done—e.g., analyses of subgroups and interactions, and sensitivity analyses.	Results (Section 3—subgroups are primary focus); N/A for other sensitivity analyses.
**Discussion**		
17	Summarize main results with respect to study objectives.	Discussion (Section 4, para 1)
18	Discuss limitations of the study, taking into account sources of potential bias or imprecision. Discuss both direction and magnitude of any potential bias.	Discussion: Limitations (Section 4, last para)
19	Discuss the generalizability (external validity) of the study results.	Discussion (Section 4, second to last para)
20	Interpret results in light of the study hypotheses, findings from other relevant studies, and other relevant explanations.	Discussion (Section 4, throughout)
**Other information**		
21	Give the source of funding and the role of the funders for the present study.	Funding (Title page/Manuscript start)
22	Report any conflicts of interest.	Conflicts of Interest (Title page/Manuscript start)
